# *HMBS* gene mutations and hydroxymethylbilane synthase activity in acute intermittent porphyria: A systematic review

**DOI:** 10.1097/MD.0000000000035144

**Published:** 2023-09-29

**Authors:** Shuang Li, Jia-Jia Lei, Bai-Xue Dong, Yi Ren, Jing Yang

**Affiliations:** a Department of the First Clinical Medical School, Shanxi Medical University, Taiyuan, China; b Department of Endocrinology, The First Hospital of Shanxi Medical University, Taiyuan, China.

**Keywords:** acute intermittent porphyria, *HMBS* activity, HMBS gene, mutation type

## Abstract

**Background:**

Acute intermittent porphyria (AIP) is caused by a partial deficiency of hydroxymethylbilane synthase and affects heme biosynthesis. Mutations in the *HMBS* gene result in *HMBS* deficiency. AIP is a rare disease, and there been insufficient studies on it. This report describes the molecular epidemiology of *HMBS* gene defects and hydroxymethylbilane synthase activity levels in classical AIP.

**Methods:**

Databases of PubMed, CNKI, and Wang Fang Database were searched for eligible studies to investigate *HMBS* gene mutations in peripheral blood samples and *HMBS* activity in erythrocytes of patients with classical AIP. Relevant studies published up to July 15, 2023, from several databases were independently searched and selected by 2 reviewers. Accuracy data and relevant information were extracted from each eligible study by 2 independent researchers and analyzed using statistical software.

**Results:**

After pooling the accuracy data from 232 patients of the 15 eligible studies, 90.5% (210/232) of AIP patients had decreased erythrocyte hydroxymethylbilane synthase activity (<70%), and 96 different mutations were identified in 232 patients, including 33 missense (34.4%), 27 splice (28.1%), 19 deletion (19.8%), 8 nonsense (8.3%), 9 insertion (9.4%) mutations. Residual enzyme activities (%) for different groups of type were expressed using mean and 95% confidence interval (95% CI): missense (51.2, 48.5–53.9), splice (57.5, 52.0–59.1), deletion (54.9, 50.7–59.1), nonsense (52.2, 44.4–60.0), insertion (53.2, 47.4–59.0), group analysis *P* = .17. Subgroups of missense mutations, domain 1 (50.2, 46.0–54.4), domain 2 (52.8, 49.1–56.4), and domain 3 (49.2, 38.3–60.0), Subgroup analysis, *P* = .62.

**Conclusion:**

Different mutation types and mutation positions are not associated with the level of hydroxymethylbilane synthase activity. Erythrocyte hydroxymethylbilane synthase activity is often reduced to half of normal in patients with AIP, and the enzyme activity assay has a high diagnostic value in AIP. AIP is highly molecularly heterogeneous, with missense mutations being the most common, followed by splice mutations. R173W and G111R are high-frequency mutations and have been found in multiple families from different countries.

## 1. Introduction

Acute intermittent porphyria (AIP, MIM # 176000) is an autosomal dominant disorder caused by a mutation in the HMBS gene that results in deficiency of the third enzyme in heme biosynthesis, hydroxymethylbilane synthase (HMBS)—also known as porphobilinogen deaminase (PBGD)—and the accumulation of the heme precursors δ-aminolaevulinic acid (δ-ALA) and porphobilinogen (PBG) in the body, resulting in a series of digestive and neuropsychiatric symptoms.^[1]^

Hydroxymethylbilane synthase is encoded by a single 11-kb gene located in chromosomal region 1 1q24.1–1 1q24.2.^[2]^ The gene contains 15 exons ranging in size from 39 to 438 bp.^[3]^ Two distinct mRNAs are produced by the alternative splicing of 2 primary transcripts arising from 2 promoters. The upstream promotor is active in all tissues, and the housekeeping transcript initiated by this promoter is encoded by exons 1 and 3–15. The other promotor lies 3 kb downstream in intron 1 and is active only in erythroid cells. Its utilization produces a transcript encoded by exons 2–15.^[4,5]^ And AIP was divided into classic AIP with decreased erythrocyte HMBS activity (95%) and nonerythroid form of AIP with normal erythrocyte HMBS activity.^[6]^

Generally, the biochemical diagnosis of AIP is based on the measurement of the urinary porphyrin precursors, δ-ALA and PBG, in combination with the determination of erythrocyte HMBS activity. Currently, the gold standard for the diagnosis of AIP is molecular analysis of the *HMBS* gene. With the development of genetic sequencing technology, >500 mutations of the *HMBS* gene have been identified in the worldwide. Previous studies of glutathione synthetase deficiency have shown that all mutations causing frameshifts, premature stop codons, or aberrant splicing were associated with moderate or severe clinical phenotypes^[7]^; a similar result was find in patients with 21-hydroxylase deficiency, large deletions with lower enzyme activity.^[8]^ Recently, there are more studies on the phenotypes of individual mutations. However, there is a lack of systematic analysis of the erythrocyte HMBS activities of classical AIP. The aim of this study was to search the erythrocyte HMBS activity of publicly published classical AIP patients with clear *HMBS* gene mutation to describe the characteristics of *HMBS* gene mutations and whether different mutation types and positions have the different level of erythrocyte HMBS activity, and to increase the understanding of the diagnostic value of erythrocyte HMBS activity.

## 2. Methods

### 2.1. Literature searching and selection of publication

A literature search was conducted independently by SL and JL on July 15, 2023. Databases including PubMed, CNKI, and Wang Fang Database were searched using the keywords “acute intermittent porphyria,” “hydroxymethylbilane synthase activity,” “porphobilinogen deaminase activity,” and “mutation,” and alternative spellings or abbreviations were also searched. Once the search results were obtained, duplicates were removed, and irrelevant studies were excluded after a careful review of the title and abstract of the publications using the following criteria. Inclusion criteria: all original studies described *HMBS* gene mutations tested on blood samples from AIP patients and all patients had erythrocyte HMBS activity measured.

Exclusion criteria:

1. not a human study;
2. not describing *HMBS* mutation;
3. not describing HMBS activity;
4. reviews, abstracts, letters to the editor, commentaries, case reports, or studies with uninterpretable data.

The full text of the remaining publications was then downloaded and carefully reviewed by 2 investigators. Publications were further excluded if:

1. with errors in the reported *HMBS* gene mutation sites;
2. no erythrocyte HMBS activity;
3. inclusion of overlapping patients;
4. uninterpretable data (difficulty in extracting precision data from the results).

Ethical approval was not required for this study because as all the data obtained and analyzed were extracted from previously published literature and not from individual patients. This systematic review was conducted according to the Preferred Reporting Items for Systematic Reviews statement checklist (PRISMA Checklist).

### 2.2. Data extraction from eligible articles

Data on patients’ age, sex, country, mutations, and erythrocyte HMBS activity were retrieved. Missing data or unclear definitions were resolved by direct contact with the authors if possible, and were otherwise considered unavailable. Two authors (SL, JL) identified the relevant original articles and extracted the data independently, while the third author (BD) checked the results. In cases of disagreement, the relevant programs were repeated until a consensus was reached among the authors.

### 2.3. Statistical analysis

Descriptive analysis was conducted to provide the overall data of mutations, mutation types, mutation positions, erythrocyte HMBS activity. One-way ANOVA was used to test for differences between groups. The continuous variables (erythrocyte HMBS activity) were presented as mean with standard deviation and with 95% confidence interval (95% CI). Counting data (mutations) were presented as percentages. For all analyses, *P* < .05 (two-tailed) was considered statistically significant. Statistical Product and Service Solutions 23.0 (SPSS, IBM Corporation, US) software was used for all analyses.

## 3. Results

### 3.1. Search results

**Figure 1.**
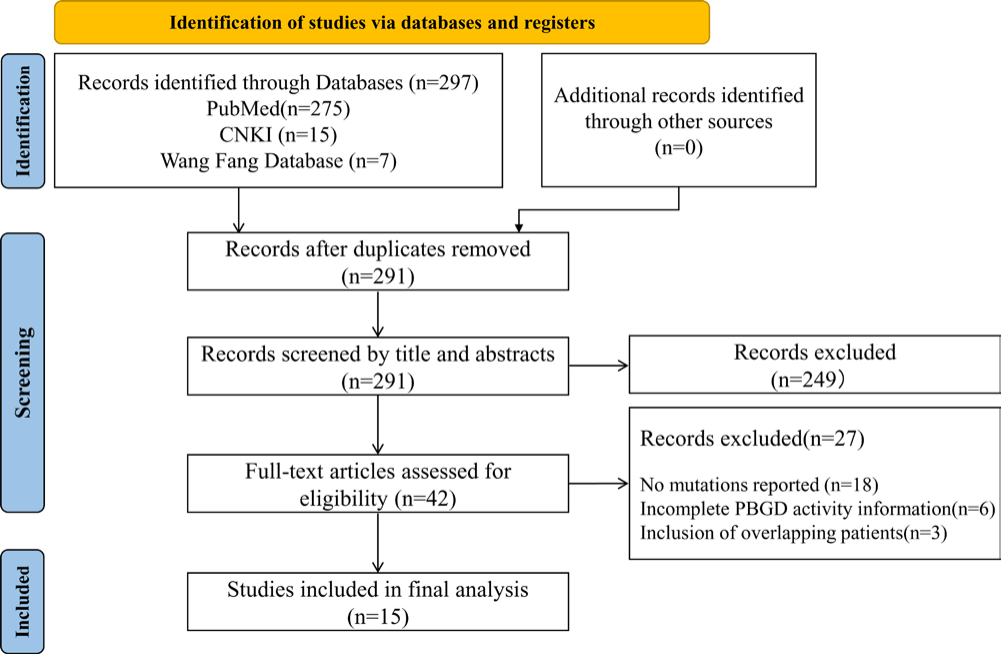
Flow chart for literature search on original articles for systematic review.

As shown in Figure 1, a total of 297 publications were identified after searching PubMed (n = 275), CNKI (n = 15), and Wang Fang Database (n = 7). After removing duplicates, titles and abstracts of 291 publications were screened. The full text of the remaining 42 studies was downloaded and evaluated, and another 27 studies were further excluded due to missing mutations, overlapping patients, and insufficient information. Finally, 15 studies were obtained for analysis.^[9–23]^

### 3.2. Review of eligible publications

Data were extracted from the remaining 15 eligible studies (see Table 1, and Supplemental Digital Content, http://links.lww.com/MD/J822, which summarizes the extracted data from the eligible studies). In the 15 eligible studies, excluded 4 individuals with the nonerythroid variant form of AIP and 11 individuals with wrong or missing information of mutation or enzyme activity, leaving 232 (contain 2 patients with homozygous mutation) cases for analysis. Enzyme activity was measured by the fluorescence method in all 15 eligible studies. And 3 studies used Denatured Gradient Gel Electrophoresis to test *HMBS* mutation in blood sample, while this number was 12 for PCR.

**Table 1:** Summary of studies about HMBS mutation status and erythrocyte HMBS activity from AIP patients.

Author, year	Sample size	No. of mutation	Detection method (mutations)	Detection method (HMBS activity)	Region
María-José et al^[9]^	55	25	PCR	Fluorescence method	Spanish
Ulbrichova et al^[10]^	26	6	PCR	Fluorescence method	Israeli
Yang et al^[11]^	24	21	PCR	Fluorescence method	Chinese
To-Figueras et al^[12]^	16	10	PCR	Fluorescence method	Spanish
Pischik et al^[13]^	20	9	PCR	Fluorescence method	Russian
Solis et al^[14]^	3	1	PCR	Fluorescence method	Spanish
Hessels et al^[15]^	3	1	PCR	Fluorescence method	Turkish
Gregor et al^[16]^	20	16	DGGE	Fluorescence method	Polish
Martinez et al^[17]^	14	13	DGGE	Fluorescence method	Italian
Ramdall et al 2000^[18]^	5	5	PCR	Fluorescence method	USA
Martinez et al^[19]^	8	7	DGGE	Fluorescence method	Italian
De Siervi et al^[20]^	22	4	PCR	Fluorescence method	Argentinean
Maeda et al^[22]^	10	1	PCR	Fluorescence method	Japanese
De Siervi et al^[23]^	12	5	PCR	Fluorescence method	Argentinean
Solis et al^[21]^	9	8	PCR	Fluorescence method	Spanish

DGGE = denaturing gradient gel electrophoresis, PCR = polymerase chain reaction.

### 3.3. Mutation spectrum

**Figure 2.**
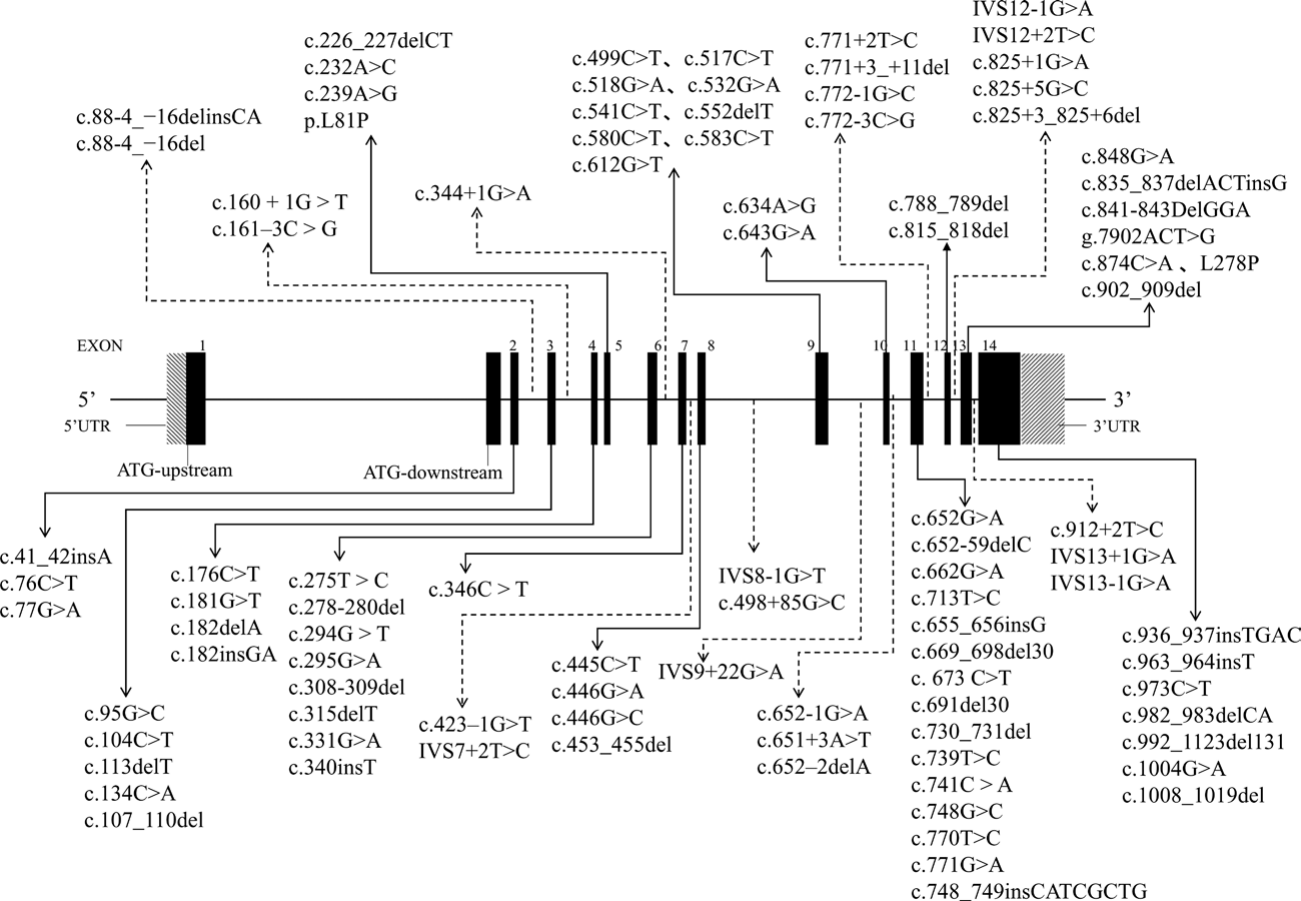
96 HMBS gene mutations spectrum in transcript (NM_000190).

In the 15 eligible studies, 232 patients were tested for *HMBS* mutation in blood sample, A total of 96 mutations were found, including 33 missense (34.4%), 27 splice (28.1%), 19 deletion (19.8%), 8 nonsense (8.3%), 9 insertion (9.4%) mutations; 71 exon mutations were found in 96 mutations; 15 of them were located in exon 11 (15.6%) and 9 in exon 9 (9.4%). There were 24 intron mutations, of which 5 (20.8%) were located in intron 11 (see Fig. 2).

### 3.4. Positions of missense mutations in the secondary structure of HMBS

**Figure 3.**
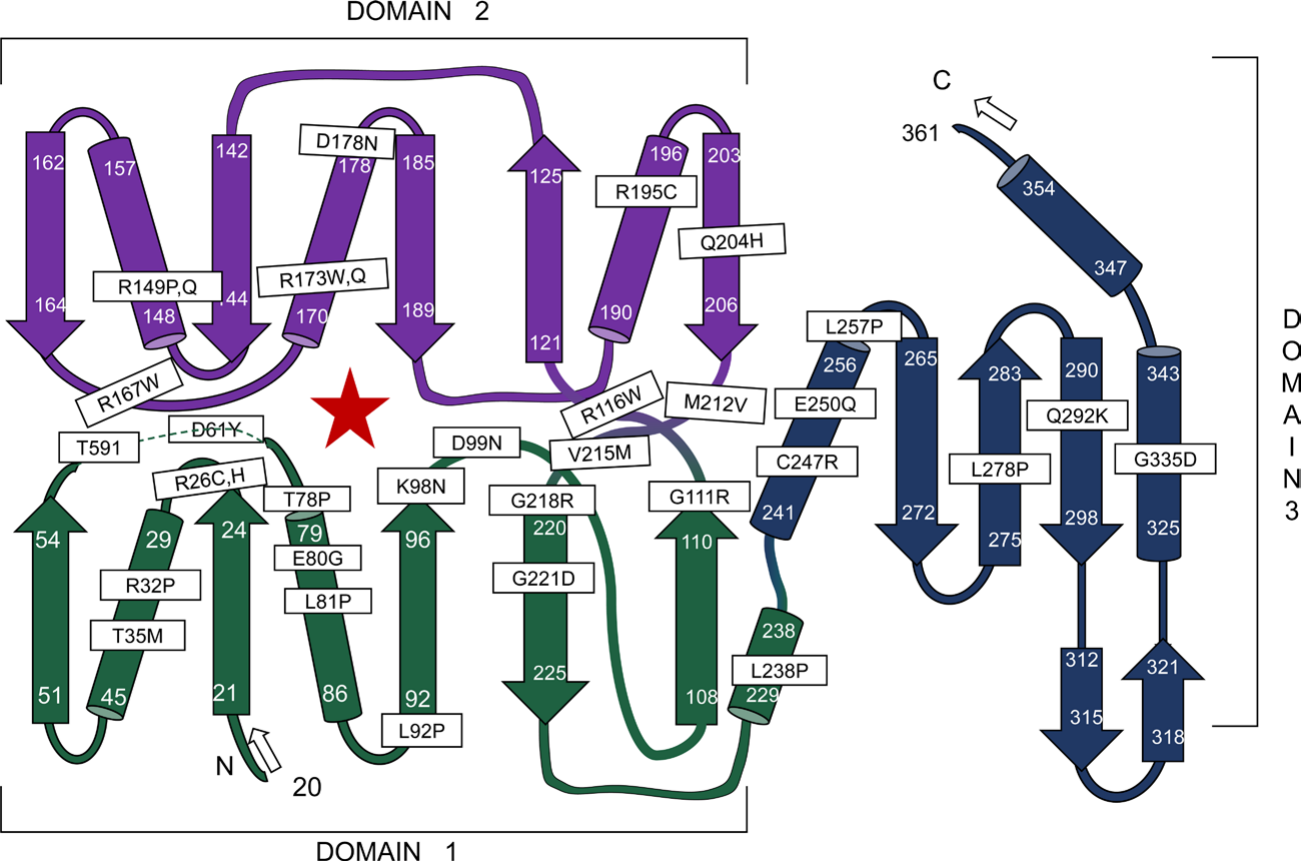
Schematic representation of HMBS secondary structural elements, based on crystal structure of *E. coli* enzyme. Helices are shown as cylinders, and the β sheets are shown as planes.^[25,26]^ The positions of the missense mutations characterized as determined by homology comparisons, are indicated; note that most of them occur near the active site and affect arginine residues.

On the basis of the *E. coli*-derived HMBS structure, it has been argued that the polypeptide chain of the human enzyme is folded into 3 domains of approximately the same size.^[24]^ The 96 mutations included 33 missense mutations, of which 17 were located in domain 1 (51.5%), 10 in domain 2 (30.3%), and 6 in domain 3 (18.2%). Figure 3 shows the secondary structure of human HMBS with the location of 33 missense mutations.

### 3.5. Model analysis of HMBS

The crystal structure of human HMBS shows that it consists 3 distinct domains of similar size. The substrate binding site is located in the cleft between domain 1 and domain 2. Domain 3 mainly stabilizes the protein conformation.^[25]^ As a cofactor of HMBS, DPM initiates the reaction in the catalysis process and interacts with several amino acids (Arg26, Ser28, Ser96, Asp99, Lys98, Ser147, Arg149, Arg150, Arg173) to form an enzyme activity center.^[3,24,27]^

**Figure 4.**
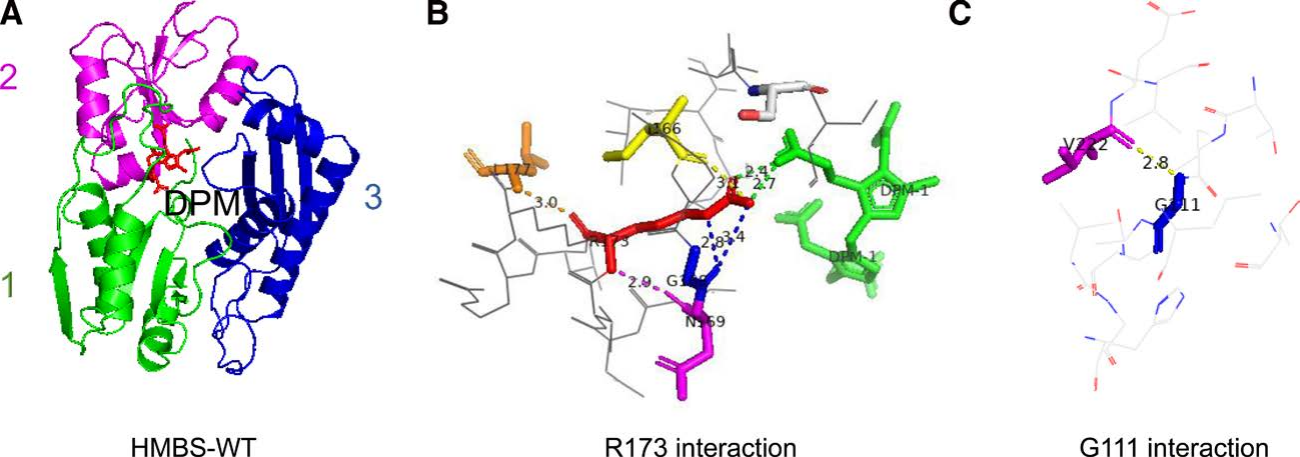
Model analysis of 2 HMBS mutations. (A) Ribbon representation of HMBS-WT. HMBS is an enzyme with 3 domains as pointed out. Domain 1 is shown in green, domain 2 is shown as in purple, domain 3 is shown as in blue, and DPM is shown as in red. (B) Dotted line representation of R173 polar contacts. R173 is shown as red, I166 is shown as yellow, G16is shown as blue, N169 is shown as purple, L177 is shown as orange, DPM is shown as green. (C) Dotted line representation of G111 polar contacts. G111 is shown as blue, V222 is shown as purple.

Based on the 15 original articles, R173W and G111R are high-frequency mutations, using the homology modeling program Swiss-Model (http://swissmodel.expasy.org) to develop a suitable model to explain the decrease in HMBS activity of the mutations, these structures are displayed by the PDB viewer software (PyMOL 2.5.4). Through the model, we can see that residue R173 interacts with residues L177, I166, G168, and N169, and it also interacts with the cofactor DPM, while the residue G111 only interacts the residue V222 (see Fig. 4). Through protein structure analysis, we can explain the pathogenesis of the 2 mutations.

### 3.6. Enzyme activity analysis in AIP

**Figure 5.**
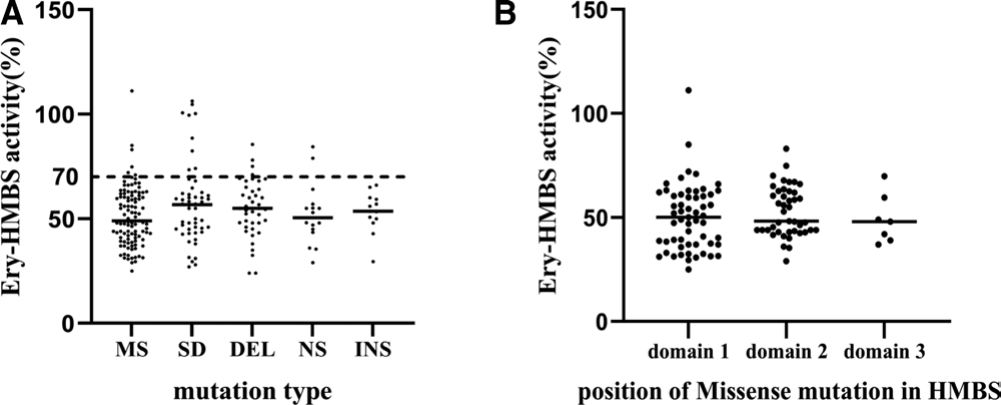
Enzyme activity analysis. AIP gene-carrier/-noncarrier status was compared for each patient, by use of erythrocyte HMBS activity and DNA analysis. Each black dots denote members of families who have the classical form of AIP. (A) HMBS activity level of different mutation types. (B) HMBS activity level of different position of missense mutation in HMBS tertiary structure. MS=missense, SD=splice defect, DEL=deletion, NS=nonsense, INS=insertion.

Based on the 15 original studies, the molecular status of 232 members (excluded 2 patients with homozygous mutation) was determined by DNA analysis and accurately classified them into 5 groups (missense, splice, nonsense, deletion, and insertion). And 33 missense mutations were classified into 3 subgroups according to the location of the tertiary structure of HMBS. The enzyme-based diagnosis was then compared with the molecular diagnosis. The HMBS activities of the members of the groups and subgroups are plotted in Figure 5. It appeared that in 90.5% of cases (210/232, Fig. 5), the AIP gene-carrier status based on the HMBS enzyme activity was confirmed by the molecular diagnosis.

One-way ANOVA was used to analyze whether erythrocyte HMBS activity (%) was different between groups and subgroups, and data are expressed as mean and 95% confidence interval (95% CI): missense (51.2, 48.5–53.9), splice (57.5, 52.0–59.1), deletion (54.9, 50.7–59.1), nonsense (52.2, 44.4–60.0), insertion (53.2, 47.4–59.0), *P* = .17 > 0.05, each group of between differences has nonstatistical significance (see Table 2). Subgroups, domain 1 (50.2, 46.0–54.4), domain 2 (52.8, 49.1–56.4) and domain 3 (49.2, 38.3–60.0), *P* = .62 > 0.05, there is no statistical significance between the groups (see Table 3).

**Table 2:** Analysis of HMBS activity between mutation types.

Type	Sample size	$\bar{\text{x}}\text{ ± s}$	95% CI	*F*	*P*
Missense	107	51.2 ± 14.1	48.5–53.9		
Splice	52	57.5 ± 19.6	52.0–59.1		
Deletion	42	54.9 ± 13.4	50.7–59.1		
Nonsense	16	52.2 ± 14.6	44.4–60.0		
Insertion	13	53.2 ± 9.6	47.4–59.0	1.635	0.166

**Table 3:** Analysis of HMBS activity between missense mutations position of HMBS.

Position	Sample size	x ± s	95% CI	*F*	*P*
Domain 1	57	50.2 ± 15.9	46.0–54.4		
Domain 2	43	52.8 ± 11.9	49.1–56.4		
Domain 3	7	49.2 ± 11.8	38.3–60.0	0.485	0.617

## 4. Discussion

AIP is an autosomal dominant disorder with a very low penetrance rate, <10% of patients have recurrent acute attacks, but acute attacks are often life-threatening.^[28]^ There are 2 types of AIP, usually the activity of HMBS in mutation carriers is about 50% of that in normal subjects, and there are no symptoms at this time, which is called the latent type; when metabolic stress, infection, smoking and drinking induce an increase in the activity of ALAS1, the rate-limiting enzyme of heme synthesis, the clinical symptoms are induced,^[28,29]^ which is called the dominant type.

Measurement of erythrocyte HMBS activity can confirm the diagnosis and it is also used to identify asymptomatic relatives. However, the enzyme activity is not always meaningful because of overlap between carrier and control values and because it is influenced by various other factors such as RBC aging and hemolysis.^[17,21]^ And there is no decrease in HMBS activity in nonerythroid AIP.^[30]^ In our study, 232 patients (included 2 patients with homozygous mutation) both are both classical form of AIP, and 90.5% of cases (210/ 232), the AIP gene-carrier status based on the HMBS enzymatic activity was confirmed by the molecular diagnosis (the HMBS activity is <70%). However, gene sequencing as the gold standard for the diagnosis of AIP has its limitations. According to incomplete statistics, about 5% of AIP families cannot detect HMBS gene mutations.^[6,31]^ It can be seen that the activity of HMBS enzyme in red blood cells as the basis for diagnosis of AIP has limitations, but it still has high diagnostic value. It can be seen that the activity of HMBS enzyme in red blood cells as the basis for diagnosis of AIP has limitations, but it still has high diagnostic value. The combination of measurement of HMBS activity with appropriate genetic testing promotes better outcomes in AIP patients and asymptomatic carriers.

Our study concluded that the mutation type and location in tertiary structure of HMBS had nonconnection with the level of erythrocyte HMBS activity of patients with AIP. The residual erythrocyte HMBS enzyme activity of patients with AIP was reduced to about half of the normal level. Currently, some mutations have been tested for enzyme activity analysis in vitro, The enzyme activity of homozygotes of most mutations in vitro expressed <5%, which is consistent with the decrease of half the residual enzyme activity of heterozygotes in vivo.^[9,10,20,21,23,24,32–38]^ However, some of the homozygotes in vitro expressed the enzyme activity close to normal, ex. c.532 G>A and c.176 C>T, the residual enzyme activity of the homozygote reached 81% in vitro, while the heterozygote residual enzyme activity was 40% in vivo,^[10,34]^ the reasons for this phenomenon need to be further explored.

The penetrance rate of AIP is low, and the heterogeneity is high. With the development of genetic sequencing technology, more and more HMBS gene mutations have been reported, and currently >500 mutations have been reported in the HGMD database (http://www.hgmd.org), with missense mutations being the most common. In our study, among the 232 patients, 96 mutations were identified, with missense mutations accounting for the largest proportion, consistent with the HGMD database. R173W mutation is the high-frequency mutation among the patients included in our study, which is consistent with multiple studies of HMBS gene analysis.^[39,40]^ Using the homology modeling program Swiss Model to develop a suitable model to explain the decrease in HMBS activity of the mutations, it was found that residue R173 interacts with residues L177, I166, G168, and N169, and it also interacts with the cofactor DPM, while residue G111 interacts with residue V222 (see Fig. 4). R173W lost these interactions and affected the combination of the cofactor with the enzyme activity center, leading to a decrease in enzyme activity. And residue R173 is located on the α2_2_ in domain 2, the mutation of this residue causes serious defects in the catalytic protein HMBS and affects the enzyme kinetics and conformational stability.^[41]^ Residue G111 is located in domain 1 β4_1_ and V222 is located in domain 1 β3_1_, the interaction between them maintains the reverse parallel structure of those 2 β-fold. G111R lost this interaction, affecting the 3-dimensional structure of the enzyme and reducing the enzyme activity.

## 5. Limitations

There are several limitations of this study. First, due to the nature of this systematic review, publication bias could not be avoided and significant heterogeneity existed in many aspects. We believe this is a suboptimal and feasible option to provide an overview of mutations and enzyme activity of AIP, when an international study is not available. Second, with the low proportion of nonerythroid AIP, this paper lacks the analysis of mutations in exon 1.

## 6. Conclusion

Acute intermittent porphyria has a high molecular heterogeneity, with missense mutations being the most common, followed by splicing mutations. The activity of erythrocyte porphyrinogen deaminase in patients with classical AIP is often decreased to half of the normal level. Determination of enzyme activity has a high diagnostic value in classical AIP. The level of erythrocyte HMBS activity is independent of the type and location of the mutation.

## Author contributions

Conceptualization: Shuang Li, Yi Ren.

Data curation: Shuang Li, Jia-Jia Lei, Bai-Xue Dong.

Formal analysis: Shuang Li, Jia-Jia Lei.

Funding acquisition: Yi Ren, Jing Yang.

Writing – original draft: Shuang Li.

Writing – review & editing: Yi Ren, Jing Yang.

## Supplementary Material


