# Supplementary material for: HMBS gene mutations and hydroxymethylbilane synthase activity in acute intermittent porphyria: A systematic review
Source: Medicine (Baltimore). 2023 Sep 29;102(39):e35144. doi: 10.1097/MD.0000000000035144 (PMC10545320; doi:10.1097/MD.0000000000035144)
Supplement: Supplementary file 1 [file medi-102-e35144-s001.docx]

| No. | Sex | Age | Country | Ex | Mutations | Type | Protein Defect | HMBS  activity  (% N) | Mean HMBS  activity  (% N) |
| --- | --- | --- | --- | --- | --- | --- | --- | --- | --- |
|  | F | 22 | Spanish | Ex 2 | c.41_42insA | INS | N14Kfs*39 | 48 | 48 |
| 1. 2 | F | 33 | Spanish |  | c.76C > T | MS | R26C | 52 | 47.1 |
| 1. 3 | F | 29 | Spanish |  | c.76C > T | MS |  | 33 |  |
|  | F | 24 | Spanish |  | c.76C > T | MS |  | 54.2 |  |
|  | F | 31 | Chinese |  | c.76C > T | MS |  | 31.2 |  |
|  | F | 37 | Chinese |  | c.76C > T | MS |  | 37.1 |  |
|  | - | - | Chinese |  | c.76C > T | MS |  | 62.5 |  |
|  | M | 34 | Italian |  | c.76C > T | MS |  | 47.5 |  |
|  | F | 37 | Argentinean |  | c.76C > T | MS |  | 62.9 |  |
|  | F | 27 | Argentinean |  | c.76C > T | MS |  | 43.4 |  |
|  | F | - | Russian |  | c.77G>A | MS | R26H | 32 | 41.1 |
|  | F | - | Russian |  | c.77G>A | MS |  | 37 |  |
|  | F | - | Russian |  | c.77G>A | MS |  | 25 |  |
|  | F | 21 | Polish |  | c.77G>A | MS |  | 40.3 |  |
|  | - | - | Chinese |  | c.77G>A | MS |  | 66.1 |  |
|  | F | 33 | Chinese |  | c.77G>A | MS |  | 56 |  |
|  | M | 37 | Chinese |  | c.77G>A | MS |  | 31.3 |  |
|  | F | 31 | Chinese | IVS 2 | c.88-4_−16del. AAGTCTCTACCCGinsCA | SD | - | 28.1 | 50.8 |
|  | - | - | Chinese |  | c.88-4_−16del. AAGTCTCTACCCGinsCA | SD | - | 73.4 |  |
|  | - | - | Chinese |  | c.88-4_−16delAAGTCTCTACCCG | SD | - | 62.6 | 53.9 |
|  | F | 34 | Chinese |  | c.88-4_−16delAAGTCTCTACCCG | SD | - | 45.2 |  |
|  | F | 21 | Israeli | Ex 3 | c.95G>C | MS | R32P | 85 | 85 |
|  | F | 29 | Argentinean |  | c.104C>T | MS | T35M | 35.8 | 35.8 |
|  | F | 36 | Chinese |  | c.113delT | DEL | V38Gfs*5 | 43.3 | 43.3 |
|  | F | 16 | Italian |  | c.134C>A | NS | S45* | 29 | 29 |
|  | F | 23 | Spanish |  | c.107_110delACAG | DEL | D36Vfs*6 | 69 | 69 |
|  | F | 28 | Spanish | IVS 3 | c.160 + 1G > T | SD | - | 55 | 55 |
|  | F | 45 | Spanish |  | c.161–3C > G | SD | - | 62 | 62 |
|  | F | 15 | Israeli | Ex 4 | c.176C>T | MS | T59I | 60.3 | 60.3 |
|  | F | 50 | Polish |  | c.181G>T | MS | D61Y | 111.2 | 111.2 |
|  | F | 33 | Italian |  | c.182delA | DEL | D61Afs*37 | 24 | 24 |
|  | F | 58 | Italian |  | c.182insGA | INS | D61Gfs*38 | 56 | 56 |
|  | F | 38 | Spanish | Ex 5 | c.226_227delCT | DEL | L76Vfs*6 | 75 | 75 |
|  | - | - | USA |  | c.232A>C | MS | T78P | 31.4 | 31.4 |
|  | - | - | USA |  | c.239A>G | MS | E80G | 48.6 | 48.6 |
|  | M | 7 | Turkish |  | L81P/L81P | MS | L81P | 3.4 | - |
|  | F | - | Turkish |  | L81P | MS |  | 62.9 |  |
|  | M | - | Turkish |  | L81P | MS |  | 50.9 |  |
|  | M | 62 | Spanish | Ex 6 | c.275T > C | MS | L92P | 53 | 53 |
|  | F | 35 | Polish |  | c.278_280delTTG | DEL | delV93 | 78 | 78 |
|  | F | 34 | Spanish |  | c.294G > T | MS | K98N | 61 | 61 |
|  | M | 67 | Italian |  | c.295G>A | MS | D99N | 62 | 62 |
|  | M | 32 | Italian |  | c.308-309delTG | DEL | A103Vfs*18 | 48 | 48 |
|  | F | 29 | Polish |  | c.315delT | DEL | P106Lfs*29 | 54.6 | 54.6 |
|  | F | 34 | Polish |  | c.331G>A | MS | G111R | 29.6 | 46.4 |
|  | F | 32 | Polish |  | c.331G>A | MS |  | 70.9 |  |
|  | F | 40 | Italian |  | c.331G>A | MS |  | 56 |  |
|  | F | 29 | Spanish |  | c.331G > A | MS |  | 47 |  |
|  | F | 61 | Spanish |  | c.331G > A | MS |  | 72 |  |
|  | F | 29 | Spanish |  | c.331G > A | MS |  | 59.7 |  |
|  | F | 19 | Spanish |  | c.331G > A | MS |  | 60.2 |  |
|  | - | - | USA |  | c.331G>A | MS |  | 37.1 |  |
|  | F | 32 | Argentinean |  | c.331G > A | MS |  | 32.5 |  |
|  | F | 20 | Argentinean |  | c.331G > A | MS |  | 55.2 |  |
|  | F | 30 | Argentinean |  | c.331G > A | MS |  | 52.4 |  |
|  | F | 27 | Argentinean |  | c.331G > A | MS |  | 40.9 |  |
|  | F | 30 | Argentinean |  | c.331G > A | MS |  | 49 |  |
|  | F | 34 | Argentinean |  | c.331G > A | MS |  | 38.4 |  |
|  | F | 34 | Argentinean |  | c.331G > A | MS |  | 37.5 |  |
|  | F | 24 | Argentinean |  | c.331G > A | MS |  | 38.8 |  |
|  | F | 17 | Argentinean |  | c.331G > A | MS |  | 39.3 |  |
|  | M | 18 | Argentinean |  | c.331G > A | MS |  | 30.8 |  |
|  | F | 24 | Argentinean |  | c.331G > A | MS |  | 32.6 |  |
|  | F | 32 | Argentinean |  | c.331G > A | MS |  | 47.4 |  |
|  | F | 32 | Spanish |  | c.340insT | INS | C114Lfs*8 | 50 | 53.8 |
|  | F | 38 | Spanish |  | c.340insT | INS |  | 57 |  |
|  | F | 21 | Spanish |  | c.340insT | INS |  | 60 |  |
|  | F | 35 | Spanish |  | c.340insT | INS |  | 53.4 |  |
|  | F | 33 | Spanish |  | c.340insT | INS |  | 59.3 |  |
|  | F | 30 | Spanish |  | g.4715insT | INS |  | 42.9 |  |
|  | F | 28 | Spanish | IVS 6 | c.344 + 1G > A | SD | - | 46 | 45.8 |
|  | F | 41 | Polish |  | c.344 + 1G > A | SD | - | 45.5 |  |
|  | F | 46 | Spanish | Ex 7 | c.346C > T | MS | R116W | 60 | 48.6 |
|  | F | 19 | Spanish |  | c.346C > T | MS |  | 48 |  |
|  | M | 25 | Spanish |  | c.346C > T | MS |  | 44 |  |
|  | F | 26 | Spanish |  | c.346C > T | MS |  | 46.4 |  |
|  | F | 24 | Spanish |  | c.346C > T | MS |  | 58.5 |  |
|  | F | 18 | Chinese |  | c.346C>T | MS |  | 35.4 |  |
|  | F | 26 | Spanish |  | c.346C>T | MS |  | 47.7 |  |
|  | F | 26 | Spanish | IVS 7 | c.423–1G > T | SD | - | 46 | 40.4 |
|  | F | 25 | Italian |  | IVS7+2T>C | SD | - | 60 | 45.5 |
|  | F | 28 | Italian |  | IVS7+2T>C | SD | - | 31 |  |
|  | F | 29 | Spanish | Ex 8 | c.445C>T | NS | R149* | 43.5 | 64 |
|  | F | 20 | Polish |  | c.445C>T | NS |  | 84.4 |  |
|  | F | 20 | Argentinean |  | c.446G>T | MS | R149L | 43.6 | 43.6 |
|  | F | 44 | Chinese |  | c.446G>C | MS | R149P | 45.4 | 55.2 |
|  | - | - | Chinese |  | c.446G>C | MS |  | 65 |  |
|  | F | 33 | Argentinean |  | c.453_455delAGC | DEL | delA151 | 32.6 | 32.6 |
|  | F | 27 | Argentinean | IVS 8 | IVS8-1G>T | SD | - | 42.7 | 43.2 |
|  | F | 23 | Argentinean |  | IVS8-1G>T | SD | - | 43.7 |  |
|  |  | - | Chinese |  | c.498+85G>C | SD | - | 64.7 | 64.7 |
|  | F | 26 | Spanish | Ex 9 | c.499C>T | MS | R167W | 56.8 | - |
|  | M | 1 | Spanish |  | c.499C>T/ c.499C>T | MS |  | 1.2 |  |
|  | F | - | Spanish |  | c.499C>T | MS |  | 55.9 |  |
|  | M | - | Spanish |  | c.499C>T | MS |  | 52.9 |  |
|  | F | 34 | Spanish |  | c.517C > T | MS | R173W | 44 | 55.9 |
|  | F | 22 | Spanish |  | c.517C > T | MS |  | 59 |  |
|  | F | 28 | Spanish |  | c.517C > T | MS |  | 55 |  |
|  | F | 23 | Spanish |  | c.517C>T | MS |  | 74.8 |  |
|  | M | 27 | Spanish |  | c.517C>T | MS |  | 67 |  |
|  | - | - | USA |  | c.517C>T | MS |  | 42.9 |  |
|  | F | - | Russian |  | c.517С>Т | MS |  | 41 |  |
|  | F | - | Russian |  | c.517С>Т | MS |  | 58 |  |
|  | F | - | Russian |  | c.517С>Т | MS |  | 43 |  |
|  | F | 27 | Spanish |  | c.517С>Т | MS |  | 61.9 |  |
|  | F | 33 | Spanish |  | c.517С>Т | MS |  | 66.1 |  |
|  | F | 20 | Spanish |  | c.517С>Т | MS |  | 67.8 |  |
|  | M | 46 | Spanish |  | c.517С>Т | MS |  | 62.7 |  |
|  | F | 18 | Spanish |  | c.517С>Т | MS |  | 47.7 |  |
|  | F | 25 | Spanish |  | c.517С>Т | MS |  | 48.3 |  |
|  | F | 45 | Polish |  | c.518G>A | MS | R173Q | 42.6 | 51.8 |
|  | F | 27 | Polish |  | c.518G>A | MS |  | 62.5 |  |
|  | F | 25 | Polish |  | c.518G>A | MS |  | 60.4 |  |
|  | F | 29 | Argentinean |  | c.518G>A | MS |  | 63.6 |  |
|  | F | 34 | Argentinean |  | c.518G>A | MS |  | 41.7 |  |
|  | F | 13 | Argentinean |  | c.518G>A | MS |  | 40 |  |
|  | F | 16 | Israeli |  | c.532G>A | MS | D178N | 36 | 40 |
|  | F | 45 | Israeli |  | c.532G>A | MS |  | 44 |  |
|  | F | 41 | Italian |  | c.541C>T | NS | Q181* | 55 | 55 |
|  | F | 53 | Polish |  | c.552delT | DEL | S184Rfs*71 | 68 | 68 |
|  | F | 31 | Italian |  | c. 580 C>T | NS | Q194* | 57 | 57 |
|  | F | - | Russian |  | c. 583G>T | MS | R195C | 43 | 43.7 |
|  | F | - | Russian |  | c. 583G>T | MS |  | 44 |  |
|  | F | - | Russian |  | c. 583G>T | MS |  | 44 |  |
|  | F | 30 | Italian |  | c. 612G>T | MS | Q204H | 29 | 56 |
|  | M | 65 | Italian |  | c. 612G>T | MS |  | 83 |  |
|  | M | 37 | Italian | IVS 9 | IVS9+22 G>A | SD | - | 38 | 38 |
|  | F | 51 | Spanish | Ex 10 | c.634A > G | MS | M212V | 70 | 68.5 |
|  | F | 34 | Spanish |  | c.634A > G | MS |  | 67 |  |
|  | F | 30 | Israeli |  | c.643G>A | MS | V215M | 55.5 | 62.1 |
|  | F | 57 | Israeli |  | c.643G>A | MS |  | 61 |  |
|  | M | 27 | Israeli |  | c.643G>A | MS |  | 69 |  |
|  | M | 25 | Israeli |  | c.643G>A | MS |  | 63 |  |
|  | F | 32 | Chinese | IVS 10 | c.652-1G>A | SD | - | 31.5 | 45.5 |
|  | - | - | Chinese |  | c.652-1G>A | SD | - | 59.4 |  |
|  | F | 17 | Spanish |  | c.651 + 3A > T | SD | - | 59 | 59 |
|  | F | 27 | Spanish |  | c.652–2delA | SD | - | 58 | 53 |
|  | F | 40 | Spanish |  | c.652–2delA | SD | - | 48 |  |
|  | F | 32 | Chinese | Ex 11 | c.662G>A | MS | G221D | 31.5 | 45.5 |
|  | - | - | Chinese |  | c.662G>A | MS |  | 59.4 |  |
|  | F | 37 | Chinese |  | c.652G>A | MS | G218R | 39.4 | 51.6 |
|  | - | - | Chinese |  | c.652G>A | MS |  | 63.7 |  |
|  | - | - | Chinese |  | c.652_59delC | SD | - | 82.4 | 82.4 |
|  | F | 29 | Chinese |  | c.655_656insG | INS | A219Gfs*32 | 50.8 | 50.8 |
|  | - | - | Chinese |  | c.713T>C | MS | L238P | 66.3 | 58.3 |
|  | F | 35 | Spanish |  | c.713T>C | MS |  | 50.2 |  |
|  | F | 43 | Spanish |  | c.669_698del30 | DEL | E223_L232del | 71 | 52.3 |
|  | F | 26 | Spanish |  | c.669_698del30 | DEL |  | 55 |  |
|  | F | 36 | Spanish |  | c.669_698del30 | DEL |  | 52 |  |
|  | F | 30 | Spanish |  | c.669_698del30 | DEL |  | 49 |  |
|  | M | 40 | Spanish |  | c.669_698del30 | DEL |  | 56 |  |
|  | F | 35 | Spanish |  | c.669_698del30 | DEL |  | 44 |  |
|  | F | 56 | Spanish |  | c.669_698del30 | DEL |  | 24 |  |
|  | F | 37 | Spanish |  | c.669_698del30 | DEL |  | 54 |  |
|  | M | 38 | Spanish |  | c.669_698del30 | DEL |  | 58 |  |
|  | F | 41 | Spanish |  | c.669_698del30 | DEL |  | 64 |  |
|  | F | 62 | Spanish |  | c.669_698del30 | DEL |  | 48 |  |
|  | F | 25 | Spanish |  | c.673C > T | NS | R225* | 64 | 48 |
|  | F | 42 | Italian |  | c. 673 C>T | NS |  | 48 |  |
|  | F | 49 | Italian |  | c. 673 C>T | NS |  | 36 |  |
|  | F | 20 | Polish |  | 673C>T | NS |  | 47 |  |
|  | F | - | Russian |  | c.673C>T | NS |  | 45 |  |
|  | F | 32 | Spanish |  | c.691del30 | DEL | D230_H239del | 85.6 | 85.6 |
|  | F | 54 | Polish |  | c.730_731delCT | DEL | L224Afs*6 | 49.3 | 52.8 |
|  | F | 45 | Israeli |  | c.730_731delCT | DEL |  | 42 |  |
|  | F | 15 | Israeli |  | c.730_731delCT | DEL |  | 46 |  |
|  | F | 43 | Italian |  | c.730_731delCT | DEL |  | 69.3 |  |
|  | F | 36 | Italian |  | c.730_731delCT | DEL |  | 38 |  |
|  | F | 19 | Spanish |  | c.730_731delCT | DEL |  | 56.8 |  |
|  | M | 56 | Spanish |  | c.730_731delCT | DEL |  | 61 |  |
|  | F | 28 | Spanish |  | c.730_731delCT | DEL |  | 60 |  |
|  | F | - | Russian |  | c.739Т>С | MS | C247R | 42 | 42 |
|  | F | 49 | Spanish |  | c.741C > A | NS | C247* | 55 | 55 |
|  | F | 44 | Spanish |  | c.748_749insCATCGCTG | INS | E250Afs*8 | 65 | 65 |
|  | F | - | Russian |  | c.748G>С | MS | E250Q | 48 | 48 |
|  | F | - | Russian |  | c.770T>C | MS | L257P | 39 | 38 |
|  | F | - | Russian |  | c.770T>C | MS |  | 37 |  |
|  | M | 37 | Spanish |  | c.771G > A | SD | - | 45 | 45 |
|  | F | 41 | Polish | IVS 11 | c.771+2T>C | SD | - | 49.3 | 49.3 |
|  | F | 57 | Spanish |  | c.771 + 3_ +11del9 | SD | - | 57 | 53.3 |
|  | F | 36 | Spanish |  | c.771 + 3_ +11del9 | SD | - | 48 |  |
|  | F | 20 | Spanish |  | c.771 + 3_ +11del9 | SD | - | 55 |  |
|  | - | - | Chinese |  | c.772-1G>C | SD | - | 68.9 | 49.3 |
|  | F | 41 | Chinese |  | c.772-1G>C | SD | - | 29.6 |  |
|  | F | - | Japanese |  | c.772-3C>G | SD |  | 100.7 | 83.7 |
|  | M | - | Japanese |  | c.772-3C>G | SD |  | 100.3 |  |
|  | M | - | Japanese |  | c.772-3C>G | SD |  | 88.5 |  |
|  | F | - | Japanese |  | c.772-3C>G | SD |  | 60.3 |  |
|  | F | - | Japanese |  | c.772-3C>G | SD |  | 104.7 |  |
|  | M | - | Japanese |  | c.772-3C>G | SD |  | 61.5 |  |
|  | M | - | Japanese |  | c.772-3C>G | SD |  | 99.5 |  |
|  | F | - | Japanese |  | c.772-3C>G | SD |  | 58.3 |  |
|  | M | - | Japanese |  | c.772-3C>G | SD |  | 106.3 |  |
|  | F | - | Japanese |  | c.772-3C>G | SD |  | 56.6 |  |
|  | M | 42 | Spanish | Ex 12 | c.788_789delTG | DEL | V263Afs*27 | 65 | 65 |
|  | F | 41 | Spanish |  | c.815_818delAGGA | DEL | K272Mfs*6 | 55 | 55 |
|  | F | 23 | Spanish | IVS 12 | c.825 + 1G > A | SD | - | 74 | 67.5 |
|  | F | 28 | Spanish |  | c.825 + 1G > A | SD | - | 61 |  |
|  | F | 38 | Argentinean |  | IVS12-1G>A | SD | - | 48.6 | 50.5 |
|  | F | 57 | Argentinean |  | IVS12-1G>A | SD | - | 43.3 |  |
|  | F | 33 | Argentinean |  | IVS12-1G>A | SD | - | 59.5 |  |
|  | F | 37 | Italian |  | IVS12+2T>C | SD | - | 43 | 46.2 |
|  | F | - | Russian |  | c.825+5G>C | SD | - | 39 | 39.5 |
|  | M | - | Russian |  | c.825+5G>C | SD | - | 40 |  |
|  | F | - | Russian |  | c.825+3_825+6del | SD | - | 47 | 57.5 |
|  | F | - | Russian |  | c.825+3_825+6del | SD | - | 68 |  |
|  | - | - | Chinese | Ex 13 | c.848G>A | DEL | W283* | 52.4 | 52.4 |
|  | M | 22 | Chinese |  | c.902_909delTCCCTGCC | DEL | V301Afs*3 | 45.2 | 45.2 |
|  | F | 24 | Spanish |  | L278P | MS | L278P | 59.6 | 59.6 |
|  | F | 28 | Spanish |  | c.835_837delACTinsG | DEL | T279Gfs*11 | 68 | 68 |
|  | F | 38 | Argentinean |  | c.841_843DelGGA | DEL | delG280 | 53.4 | 54.3 |
|  | M | 8 | Argentinean |  | c.841_843DelGGA | DEL |  | 61.3 |  |
|  | F | 10 | Argentinean |  | c.841_843DelGGA | DEL |  | 48.2 |  |
|  | F | 27 | Spanish |  | g.7902ACT>G | INS | C261Efs*29 | 66 | 66 |
|  | M | 15 | Italian |  | c.874C>A | MS | Q292K | 49 | 49 |
|  | F | 39 | Spanish | IVS 13 | c.912 + 2T > C | SD | - | 60 | 60 |
|  | F | 31 | Spanish |  | IVS 13+1G>A | SD | - | 27 | 27 |
|  | F | 16 | Spanish |  | c.913-1G > A | SD | - | 50 | 53.5 |
|  | F | 38 | Italian |  | IVS13-1G>A | SD | - | 57 |  |
|  | F | 32 | Chinese | Ex 14 | c.963_964insT | INS | N322* | 29.6 | 29.6 |
|  | F | 21 | Chinese |  | c.936_937insTGAC | INS | P313* | 53.7 | 53.7 |
|  | M | 38 | Spanish |  | c.973C > T | NS | R325* | 53 | 54.2 |
|  | F | 30 | Spanish |  | c.973C > T | NS |  | 78.9 |  |
|  | F | 40 | Chinese |  | c.973C>T | NS |  | 55.2 |  |
|  | F | 15 | Chinese |  | c.973C>T | NS |  | 35.6 |  |
|  | - | - | Chinese |  | c.973C>T | NS |  | 48.2 |  |
|  | F | 29 | Polish |  | c.982_983delCA | DEL | Q328Vfs*30 | 63.1 | 59.1 |
|  | M | 30 | Israeli |  | c.982_984delCA | DEL |  | 55 |  |
|  | F | 35 | Polish |  | c.992_1123del131 | DEL | Q332H361del | 60 | 60 |
|  | F | 36 | Polish |  | c.1004G>A | INS | G335D | 69.8 | 69.8 |
|  | - | - | Chinese |  | c.1008_1019del CAGCCTGGCCAA | DEL | S337_N340del | 69 | 52 |
|  | F | 23 | Chinese |  | c.1008_1019del CAGCCTGGCCAA | DEL |  | 35 |  |
